# Tailoring the thermal and thermomechanical characteristics of novel MAX phase boron composites in high-temperature applications†

**DOI:** 10.1039/d5na00063g

**Published:** 2025-03-26

**Authors:** Md. Shahinoor Alam, Mohammad Asaduzzaman Chowdhury, Md. Saiful Islam, Md. Moynul Islam, Tasmina Khandaker, M. A. Gafur, Dipa Islam

**Affiliations:** a Department of Mechanical Engineering, Dhaka University of Engineering and Technology, Gazipur Gazipur 1707 Bangladesh majshahin4282@gmail.com; b Department of Chemistry, Bangladesh Army University of Engineering and Technology, Qadirabad Cantonment Natore-6431 Bangladesh; c Bangladesh Council of Scientific and Industrial Research (BCSIR) Dhanmondi Dhaka-1205 Bangladesh

## Abstract

MAX phase composites are gaining great attention for their excellent attributes in high-temperature applications like aerospace, energy, and nuclear industries. However, tailoring their thermal and thermomechanical properties for better performance at elevated temperatures remains a significant challenge. Therefore, the aim of this study is to synthesize novel MAX phase boron (B) composites for high-temperature applications. Titanium aluminum nitride (Ti_4_AlN_3_) and titanium aluminum carbide (Ti_3_AlC_2_) MAX phase reinforced B composites were prepared using the hot-pressing method at three different sintering temperatures: 1050 °C, 1250 °C, and 1325 °C. Thermal stability, thermal conductivity and thermomechanical properties of MAX phase composites were investigated through thermogravimetric analysis (TGA), hot disk method, and thermomechanical analyzer (TMA). The results reveal that thermal stability and thermal conductivity increased with rising sintering temperatures for both MAX composites. This is because higher sintering temperatures enhance atomic diffusion, densification, and particle bonding, leading to improved thermal stability and thermal conductivity of the composite. Moreover, the thermal stability of the Ti_4_AlN_3_ composite is higher than that of the Ti_3_AlC_2_ composites. At 1325 °C sintering, Ti_3_AlC_2_ composites remain stable up to 600 °C with 1.4% weight loss, while the Ti_4_AlN_3_ composite shows better stability up to 700 °C with only 0.6% weight loss. These MAX phase composites also show varying coefficients of thermal expansion (CTEs) at different temperature ranges, indicating that their thermal expansion properties are highly dependent on sintering temperatures. Both MAX composites exhibit lower overall CTEs at higher sintering temperatures, suggesting enhanced thermal stability. The negative CTEs at higher sintering temperatures in both materials suggest unusual thermal behavior, possibly due to phase transitions, secondary phase formation, or microstructural changes. These findings offer valuable insights into their thermal stability and decomposition characteristics, which are vital for high-temperature applications in electronics, optoelectronics, and semiconductor devices.

## Abstract 2

The thermal stability and decomposition patterns of Ti_4_AlN_3_ and Ti_3_AlC_2_ MAX phase boron composites are prerequisites for high temperature applications in electronics, optoelectronics and semiconductor devices.

## Graphical abstract

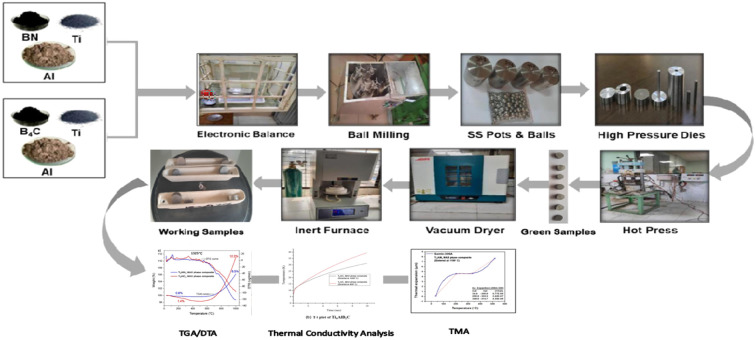

## 1 Introduction

MAX phases are a family of ternary carbides and nitrides with the general formula *M*_*n*+1_AX_*n*_, where M is an early transition metal, A is an element from groups 13–16, and X is carbon or nitrogen, renowned for their unique combination of metallic and ceramic properties.^1^ These materials exhibit high thermal conductivity and excellent thermal shock resistance, and maintain mechanical stability at elevated temperatures. Recent studies have tried to determine and improve these composites' thermal conductivity so that they can be used to their full potential in high-temperature applications.^2^ Because of their unique structure, MAX phase composites have received a lot of attention for having great thermal properties that combine the best parts of metals and ceramics. The thermal conductivity of these materials, particularly in high-temperature environments, is a critical factor influencing their application in various advanced technologies.

One important physical characteristic of MAX phases for high-temperature applications is their thermal conductivity. These materials have thermal conductivities ranging from 12 to 60 W m^−1^ K^−1^ at room temperature, noted for their efficient thermal conduction.^3^ The temperature gradient and the intrinsic thermal conductivity of these materials both affect heat transport within them. Heat is transported through MAX phases by charge carriers (electrons) and lattice vibrations (phonons), which spread and collide randomly. In metals, heat conduction is primarily electronic due to the abundance of conduction electrons, but impurities acting as scattering centers can reduce overall heat conductivity (*κ*_th_ = *κ*_e_ + *κ*_ph_).^4^ In insulators, where conduction electrons are few, phonons are the main heat carriers, and phonon scattering caused by porosity and other lattice flaws decreases lattice thermal conductivity. At high temperatures, phonon–phonon scattering, particularly anharmonic Umklapp processes, significantly boosts thermal resistance.^5^ However, the lighter aluminum (Al) atoms in MAX phases scatter fewer phonons, which raises the lattice thermal conductivity.

The thermal conductivity of amorphous boron is another important property that influences its applications in various high-temperature and electronic materials.^6,7^ Unlike crystalline B, which typically exhibits high thermal conductivity due to its well-ordered atomic structure, amorphous boron has a disordered atomic arrangement that leads to lower thermal conductivity. This reduced thermal conductivity is primarily due to the scattering of phonons, which are the primary carriers of heat in non-metallic solids. In amorphous materials, the lack of long-range order disrupts the phonon transport, resulting in higher thermal resistance. Despite this, amorphous boron can still be useful in applications requiring thermal insulation or controlled thermal properties. For instance, its lower thermal conductivity makes it a potential candidate for use in thermoelectric materials, where the ability to maintain a temperature gradient is crucial. Understanding the thermal conductivity of amorphous boron is thus essential for optimizing its performance in various technological applications.^8^ Kalidasan *et al.* synthesized MXene-based phase change material (PCM) nanocomposites using a binary eutectic PCM blend of sodium sulfate decahydrate (SSD) and sodium phosphate dibasic dodecahydrate (SPDD) in varying weight fractions. The fabrication process followed a two-step technique. The resulting nanocomposite PCM demonstrated excellent chemical stability, high optical absorption (1.1), low transmissibility (10.2%), reliable thermal conductivity (0.621 W m^−1^ K^−1^), enhanced melting enthalpy (161.2 J g^−1^), and significant cycle stability.^9^ The M_3_AX_2_ and M_4_AX_3_ families of layered ternary carbides and nitrides, where M is an early transition metal, A is an A-group element, and X is either carbon (C) or nitrogen (N), are closely related to this ternary combination.^7,8^ These materials exhibit unique properties, including high thermal and electrical conductivities, ranging from 10 to 40 W m^−1^ K^−1^ and 0.5 to 14 S m^−1^ respectively.^10^ Their Vickers hardness ratings range from 2.5 to 5 GPa, indicating that they are relatively soft and easy to machine. Instead of melting, these materials typically decompose peritectically into the A-group element and the transition metal carbide or nitride.

While MAX phases like Ti_4_AlN_3_ and Ti_3_AlC_2_ show promise for extreme-condition applications, their brittleness limits practical use.^10^ Incorporating boron, known for its hardness and strong covalent bonding, is proposed to improve their mechanical and electronic properties.^11^ However, research on boron-reinforcement MAX phase composites remains limited, with challenges in achieving uniform dispersion and understanding phase interactions. In this study, *in situ* Ti_3_AlC_2_ and Ti_4_AlN_3_ MAX phase-reinforced boron-based composites were synthesized *via* hot pressing and inert sintering, and their thermal and thermomechanical properties were systematically investigated. Previous studies utilizing SEM, EDS, FE-SEM, AFM, and XRD reported significant transformations in surface morphology and phase composition with increasing sintering temperature. These changes resulted in a reduction in dislocation density and microstrain, enhancing structural integrity.^12,13^ Moreover, XRD and FE-SEM analyses confirmed the presence of MAX phases, secondary phases, and amorphous boron.^12^ The complex electronic structure of the MAX phases, characterized by multiple elements in various oxidation states and bonding configurations, resulted in non-linear electrical responses, including negative capacitance. Negative capacitance arises when the effective capacitance of the system appears to have a negative value due to specific charge dynamics, often influenced by the interplay between the layered structure of MAX phases and the applied electric field. For Ti_3_AlC_2_, inductance remained unchanged up to 1325 °C, and the temperature dependency of impedance exhibited a trend similar to that of inductance for both compounds.^14^ The robustness of these composites, formed at elevated temperatures, underscores their suitability for demanding high-temperature applications such as aerospace, automotive, and electronics industries, where thermal stability is a critical requirement. The onset temperatures for decomposition provide vital information for the processing and manufacturing of these nanocomposites. Knowing these temperatures helps to avoid critical points during production, thereby ensuring the integrity and performance of the materials. This is particularly important in industries where maintaining material performance at high temperatures is essential.

The TGA and DTG analyses facilitate the optimization of these processes, ensuring that the materials perform optimally under the desired conditions. For example, in catalytic converters, the ability to withstand high temperatures and oxidize in a controlled manner can significantly enhance efficiency and durability. For instance, in re-entry vehicles, materials that degrade in a controlled manner can protect the spacecraft from intense heat.^15^ Again, the coefficient of thermal expansion (CTE) for MAX-phase composites is an important property that affects their performance in various applications, particularly in high-temperature environments. The ability to control and predict the CTE through sintering temperature and understanding the underlying mechanisms allows for the design of materials with tailored thermal properties for specific applications. Ti_4_AlN_3_ and Ti_3_AlC_2_ MAX phase composites were selected for this study due to their distinct structural and compositional differences, which influence their thermal and mechanical properties. Ti_4_AlN_3_ (a nitride-based MAX phase) and Ti_3_AlC_2_ (a carbide-based MAX phase) vary in the number of atomic layers, affecting their stability and conductivity. Investigating these two compositions provides valuable insights into how different MAX phases respond to high-temperature environments, making them suitable for advanced applications.^16^ With this backdrop, the novelty of this work lies in its comprehensive thermal analysis of Ti_4_AlN_3_ and Ti_3_AlC_2_ MAX-phase composites, which provides valuable insights into their thermal stability, decomposition mechanisms, and temperature-dependent properties.

## 2 Materials and methodology

### 2.1 Materials

Necessary materials and chemicals used in this research work for the synthesis of intended MAX phase composites were in the powder form. These materials were purchased from different sources. Among these titanium (Ti) powder (Thermo Scientific, –325 mesh, 99.5% purity, CAS # 7440-32-6), aluminum (Al) powder (Thermo Scientific, −100 + 325 mesh, 99.5% purity, CAS # 7429-90-5), and boron nitride (BN) (Sigma-Aldrich, 99% purity, particle size: ≤150 nm, CAS # 10043-11-5) were purchased from USA. Other important chemicals like boron carbide (B_4_C) (Sigma-Aldrich, 98% purity, particle size: −200 mesh, CAS # 12069-32-8) and acetone (Merck KGaA, K42588114 838 1.00014.1000), utilized for this research work, were purchased from Germany. All other related materials like Flash Dry Silver Paint and Thinner for Flash Dry Silver Paint (SPi supplies, USA, Lot No.: 1180905) and Tungsten Carbide (WC) Powder (Inframat Advanced Materials, 99.9%, Lot: IAM7020WC6, Catalog: 74R-0606) were collected from the local market and utilized without additional purification.

### 2.2 Preparation of working samples using hot pressing and an inert sintering process

**Fig. 1:**
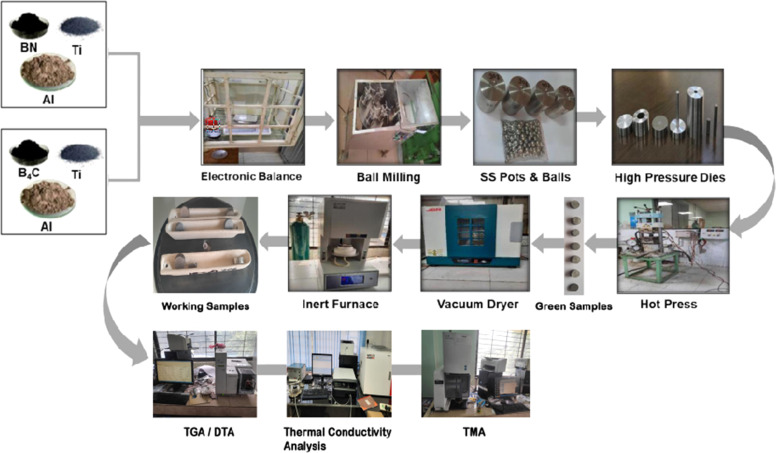
Synthesis process of working samples and its experimental techniques.

In this research work, the inert sintering process after mechanical alloying (ball milling) and hot pressing is being used. The pictorial flow diagram of the synthesis process of working samples and its experimental techniques are illustrated in Fig. 1.

Ti, Al and B_4_C or BN particles are mixed in a planetary vertical ball milling machine for the highest degree of fineness and mixing. The ball milling machine is operated in a 5 : 1 ratio of sample and ball by weight for 4 hours at 300 rpm and 22.3 Hz (frequency). The hydraulic hot press is used to compress the particles at 300–380 °C temperature and 20–30 MPa pressure with a high pressure die and mold for obtaining the desired shape of pre-bonding MAX phase reinforced composites named as green samples. The green samples are inserted into a dryer for 24 hours at 80 °C before sintering. The dried samples are taken into the inert furnace and sintered in alumina crucibles with heating and cooling rates of 5 °C min^−1^ at various temperatures ranging from 950 °C to 1325 °C at 2 hours holding time. After cooling and required preparations, the sample composites were tested and analyzed using an EXSTAR 6000 TGA/DTA6300, SII, Japan; TECHNOFOUR Conductivity Meter, Type 979, India and EXSTAR 6000 TMA/SS6300, SII, NANOTECHNOLOGY, Japan.

## 3 Results and discussion

### 3.1 Thermogravimetric analysis

#### 3.1.1 Thermal stability and oxidation reactions

**Fig. 2:**
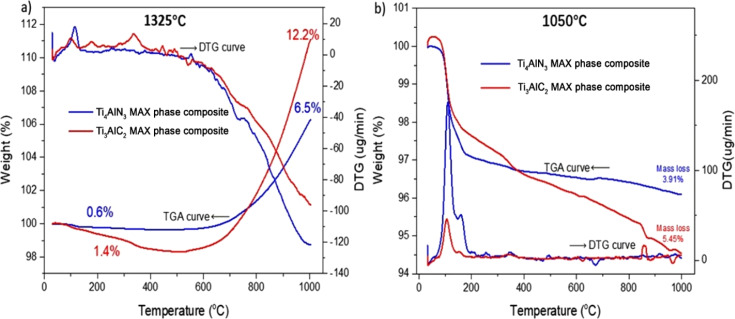
Typical thermogravimetric curves of Ti_4_AlN_3_ and Ti_3_AlC_2_ MAX phase composite samples sintered at different temperature: (a) TGA (weight variation) and DTG (derivative of weight variation) thermograms of MAX phase composite samples sintered at 1325 °C; (b) TGA (weight variation) and DTG (derivative of weight variation) thermograms of MAX phase composite samples sintered at 1050 °C.

Thermogravimetric analysis (TGA) at 1000 °C offers essential insights into the thermal stability and decomposition behavior of materials. When studying Ti_4_AlN_3_ and Ti_3_AlC_2_ MAX phase composites subjected to sintering at 1325 °C and 1050 °C as shown in Fig. 2(a) and b, TGA helps to clarify the differences in mass loss profiles and thermal responses between these two conditions. Sintering at 1325 °C generally leads to higher densification and fewer residual volatile compounds than at 1050 °C, as the higher temperature promotes more complete sintering and removal of impurities. As a result, TGA curves for samples sintered at 1325 °C typically exhibit less mass loss at high temperatures compared to those sintered at 1050 °C, indicating better thermal stability and potentially different phases or microstructural properties. This comparative TGA analysis is valuable for optimizing sintering processes and tailoring material properties for specific applications.^2^

The TGA and DTG thermograms for the MAX phase composites are presented in Fig. 2(a) and (b) which highlights important thermal characteristics. Notably, the Ti_4_AlN_3_ MAX phase composite exhibits considerable resistance to oxidation up to 700 °C in the samples sintered at 1325 °C and mass gain rates (6.5%) observed at around 1000 °C temperature, which is an indication mark of oxidation reactions. In contrast, the Ti_3_AlC_2_ MAX phase composite demonstrates a significantly higher mass gain rate (12.2%) than that of the Ti_4_AlN_3_ MAX phase composite. This difference of resistance to oxidation implies that with high-temperature sintering, the Ti_4_AlN_3_ MAX phase composite maintains its structural integrity more effectively than the Ti_3_AlC_2_ MAX phase composite under high-temperature conditions, particularly at 1000 °C.^17^

TGA conducted at 1000 °C provides crucial insights into the thermal stability and decomposition characteristics of materials. When examining Ti_4_AlN_3_ and Ti_3_AlC_2_ MAX phase composites subjected to sintering temperatures of 1325 °C and 1050 °C, TGA helps to elucidate the differences in mass loss profiles and thermal behaviors between these two conditions.^8,17^ The Ti_4_AlN_3_ MAX phase composite shows a minimal mass loss of 0.6%, while the Ti_3_AlC_2_ MAX phase composite has a slightly higher mass loss of 1.4% sintering at 1325 °C as shown in Fig. 2(a). For the materials sintered at 1050 °C as shown in Fig. 2(b), both Ti_4_AlN_3_ and Ti_3_AlC_2_ MAX phase composites exhibit greater mass losses compared to their counterparts sintered at 1325 °C. In this case, Ti_4_AlN_3_ MAX phase composite shows a total mass loss of 3.91%, whereas the Ti_3_AlC_2_ MAX phase composite displays a higher mass loss of 5.45%. The DTG curves of the samples sintered at lower temperature (1050 °C) reveal more pronounced peaks, indicating higher decomposition rates and the presence of more volatile components. On the other hand, at higher sintering temperatures (1325 °C) it leads to more thermally stable composites with lower mass loss and fewer impurities. The comparative analysis using TGA thus provides valuable information for optimizing sintering processes and tailoring material properties for specific applications. The significant oxidation reactions and mass gain rates observed at 1000 °C with the high temperature sintered sample at 1325 °C underscore the robustness of these materials, particularly the Ti_4_AlN_3_ MAX phase composite, which maintains its structural integrity better than the other MAX phase composites.^2^

#### 3.1.2 Temperature-dependent behavior

**Fig. 3:**
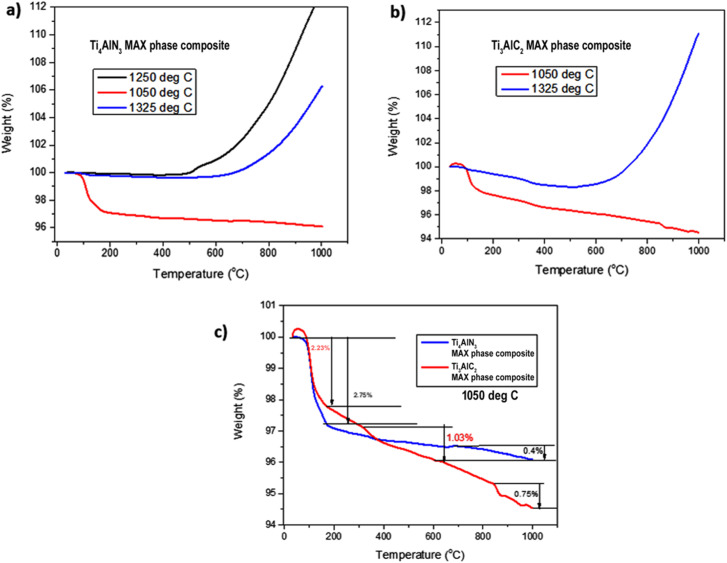
Comparison of TGA values of (a) Ti_4_AlN_3_ MAX phase composite samples sintered at 1050 °C, 1250 °C and 1325 °C temperatures; (b) Ti_3_AlC_2_ MAX phase composite samples sintered at 1050 °C and 1325 °C temperatures; (c) Ti_4_AlN_3_ and Ti_3_AlC_2_ MAX phase composite samples sintered at 1050 °C.

The thermogravimetric analysis (TGA) profiles of Ti_4_AlN_3_ and Ti_3_AlC_2_ MAX phase composites sintered up to 1325 °C as shown in Fig. 3(a)–(c) provide crucial insights into their thermal behaviors and stability. The identification of critical temperatures of samples sintered at 1050 °C, 1250 °C and 1325 °C highlights key points of notable changes in weight loss or stability for both materials. Understanding these temperature-dependent behaviors is paramount for tailoring the applications of these materials to ensure optimal performance and integrity, particularly in high-temperature environments.

Specifically, observations on the sample sintered at 1050 °C as shown in Fig. 3(c) indicate the onset of thermal decomposition for both materials. But the decomposition processes for the sample material of the Ti_4_AlN_3_ MAX phase composite (sintered at 1250 °C) are not significant and maintain its thermal stability up to 500 °C, leading to accelerated rates of weight gain after this temperature. As sintering temperatures reach 1325 °C, the materials approach their thermal stability limits up to 700 °C with the Ti_3_AlC_2_ MAX phase composite displaying more significant weight gain (12.2%) compared to the Ti_4_AlN_3_ MAX phase composite (6.5%). The diffusion of the A-site element (Al) in Ti_4_AlN_3_ and Ti_3_AlC_2_ plays a critical role in their thermal stability and high-temperature behavior. Previous studies have been carried out by Tang *et al.* and Zhang *et al.* and it was shown that A-site elements in MAX phases, such as Sn in Ti_2_SnC, exhibit significant diffusion under thermal activation, leading to phase instability and vacancy formation.^18,19^ Similarly, in Ti_4_AlN_3_ and Ti_3_AlC_2_, Al diffusion likely occurs through vacancy migration or grain boundary movement, with the extent of diffusion influenced by the strength of Ti–N and Ti–C bonds. Ti_4_AlN_3_, with its stronger Ti–N bonds, may exhibit lower Al mobility and greater thermal stability, as indicated by its more controlled mass loss. Conversely, Ti_3_AlC_2_, with its more metallic Ti–C bonding, allows for increased Al diffusion, contributing to its higher weight gain (12.2%) compared to Ti_4_AlN_3_ (6.5%) at elevated temperatures. These diffusion processes can lead to oxidation, structural transformations, or even MXene-like phase evolution, making the study of A-site mobility crucial for understanding the thermal performance of these materials in high-temperature applications.

This temperature sensitivity is crucial for comprehending the behavior of these materials under extreme conditions and for optimizing their applications based on their thermal response profiles, thereby ensuring their effectiveness and durability in high-temperature environments.

#### 3.1.3 Critical temperature points and decomposition behavior

##### 3.1.3.1 Sample sintered at around 1050 °C

The TGA curve for the Ti_3_AlC_2_ MAX phase composite initially shows a notable weight increase as shown in Fig. 3(b) and (c), which could be due to surface oxidation or adsorption processes. This initial weight gain is significant as it points to the material's interaction with the surrounding environment before the onset of decomposition.^20,21^ Such interactions are crucial for understanding how the material behaves when first exposed to high temperatures.^22^ As the temperature rises, this weight gain is followed by a marked weight loss, aligning with the overall decomposition pattern. This suggests that after an initial phase of oxidation, the Ti_3_AlC_2_ MAX phase composite undergoes rapid decomposition. Both Ti_4_AlN_3_ and Ti_3_AlC_2_ MAX phase composites begin to exhibit initial signs of thermal decomposition at approximately 100 °C as shown in Fig. 3(c). This early stage of weight loss can be attributed to the evaporation of adsorbed water or other volatile substances that might be present on the surface of these MAX phase composites.

##### 3.1.3.2 Sample sintered at around 1250 °C and 1325 °C

The Ti_4_AlN_3_ MAX phase composite sample sintered at 1250 °C shows a pronounced increase in weight starting around 500 °C, indicating significant oxidation as shown in Fig. 3(a). But the sample (Ti_4_AlN_3_ MAX phase composite) sintered at 1325 °C shows its thermal stability limit up to 700 °C. Then the weight gain continues, peaking close to 6.5% at 1000 °C reflecting increased oxidation reactions. Again, sintering at 1325 °C, the Ti_3_AlC_2_ MAX phase composite approaches its thermal stability limit up to 700 °C, showing more substantial weight gain (12.2%) compared to the Ti_4_AlN_3_ MAX phase composite (6.5%). The more stable mass loss stages for samples sintered at 1050 °C as shown in Fig. 3(c) for the Ti_4_AlN_3_ MAX phase composite (2.75% and 0.4%) than that of the Ti_3_AlC_2_ MAX phase composite (2.23%, 1.03% and 0.75%) suggests a gradual degradation of the material's structure, maintaining a more stable thermal profile.

The mass loss and gain percentages at various stages provide a detailed breakdown of the decomposition process and adsorption for these MAX phase composites. For the Ti_4_AlN_3_ MAX phase composite, the recorded mass loss and gain percentages of 0.6% and 6.5% and for the Ti_3_AlC_2_ MAX phase composite those of 1.4% and 12.2% respectively at different stages as shown in Fig. 2(a) suggest multiple phases of weight reduction, adsorption and stability. These stages likely correspond to initial decomposition, stability and surface oxidation, followed by more substantial internal decomposition processes. The complex decomposition pattern reflects the multifaceted nature of Ti_4_AlN_3_ and Ti_3_AlC_2_ MAX phase composites' thermal degradation, involving both surface and bulk material changes. The lower and more stable mass loss percentages indicate a less aggressive decomposition process, confirming its higher thermal stability. These stages likely represent a gradual release of surface-bound elements or minor phase changes, followed by a slow and steady degradation of the material's structure.^23^

### 3.2 Thermal conductivity analysis

#### 3.2.1 Thermal conductivity analysis of Ti_4_AlN_3_ and Ti_3_AlC_2_ MAX phase composites

**Fig. 4:**
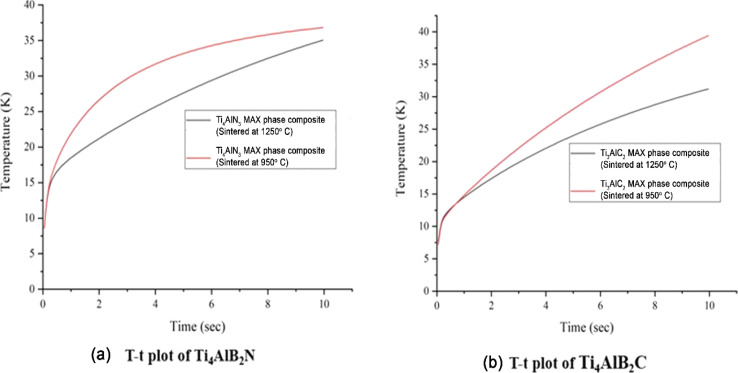
*T*–*t* plots at different sintering temperatures of (a) the Ti_4_AlN_3_ MAX phase composite and (b) Ti_3_AlC_2_ MAX phase composite samples.

Fig. 4 shows the temperature rise over time (*T*–*t* plots) for Ti_4_AlN_3_ and Ti_3_AlC_2_ MAX phase composite samples sintered at 950 °C and 1250 °C. The plots indicate that for both composites, the samples sintered at 950 °C exhibit a more rapid and higher temperature rise compared to those sintered at 1250 °C, suggesting lower thermal conductivity for the samples sintered at the lower temperature. Ti_4_AlN_3_ MAX phase composite samples sintered at 950 °C exhibit a faster and higher temperature rise compared to those sintered at 1250 °C, indicating higher thermal resistance.

Similarly, Ti_3_AlC_2_ MAX phase composite samples show a more rapid and higher temperature rise when sintered at 950 °C, suggesting the same trend. But Ti_3_AlC_2_ MAX phase composite samples display higher conductivity than that of Ti_4_AlN_3_ MAX phase composite samples when sintered at higher temperature (1250 °C).

To gain insights into the thermal conductivity mechanisms, it is needed to understand the microstructural changes that occur during the sintering process at different temperatures. Sintering at 1250 °C might result in a denser microstructure with fewer grain boundaries, which could facilitate more efficient heat transfer. On the other hand, sintering at 950 °C might introduce more grain growth and porosity, increasing thermal resistance and reducing overall thermal conductivity. These microstructural differences significantly impact the material's ability to conduct heat. Another aspect to consider is the role of grain boundaries in thermal conductivity. Grain boundaries can act as barriers to heat flow, scattering phonons and thus reducing the material's ability to conduct heat. At higher sintering temperatures, the increase in grain size could lead to a reduction in the number of grain boundaries, potentially enhancing thermal conductivity. However, if the grain boundaries become more pronounced or if porosity increases, this can counteract the benefits of reduced grain boundaries, leading to an overall decrease in thermal conductivity.

The intrinsic properties of Ti_4_AlN_3_ and Ti_3_AlC_2_ MAX phase composites also play a crucial role in their thermal behavior. Both of these materials belong to the MAX phase family, which is known for its unique combination of metallic and ceramic properties.^13,16^ These properties contribute to the materials' ability to conduct heat and their overall thermal stability. However, the specific composition and atomic structure can lead to variations in thermal conductivity. For example, the presence of different elements such as nitrogen or carbon can influence the bonding strength and the ability of the material to transfer heat. Both Ti_4_AlN_3_ and Ti_3_AlC_2_ MAX phase composites exhibit similar patterns in temperature rise, with specific values and rates varying due to intrinsic material properties. Therefore, to analyze the thermal properties of Ti_4_AlN_3_ and Ti_3_AlC_2_ MAX phase composites at two different temperatures (950 °C and 1250 °C), it is imperative to examine their thermal conductivity (TC), thermal diffusivity (TD), and specific heat (SH). These properties as shown in Table 1 provide essential insights into how these materials handle heat, which is critical for their potential applications in various industries. Understanding these mechanisms allows for optimization of these materials for various applications, emphasizing the importance of tailoring the sintering process. In practical applications, materials with higher thermal conductivity are crucial for efficient heat transfer in systems like thermal management, heat exchangers, and electronic devices. Thus, adjusting the sintering temperature is a valuable tool for optimizing the thermal properties of these composites for specific uses.

**Table 1:** Thermal poperties of Ti_4_AlN_3_ and Ti_3_AlC_2_ MAX phase composites at different sintering temperatures

Sample composites	950 °C			1250 °C		
	Thermal conductivity (TC), W m^−1^ K^−1^	Thermal diffusivity (TD), mm^2^ s^−1^	Specific heat (SH), MJ m^−3^ K^−1^	Thermal conductivity (TC), W m^−1^ K^−1^	Thermal diffusivity (TD), mm^2^ s^−1^	Specific heat (SH), MJ m^−3^ K^−1^
Ti_4_AlN_3_ MAX phase composites	0.203	0.028	0.2352	0.303	0.030	9.002
Ti_3_AlC_2_ MAX phase composites	0.208	0.030	0.2889	0.329	0.040	8.601

##### 3.2.1.1 Thermal conductivity of the Ti_4_AlN_3_ MAX phase composite

The thermal conductivity of the Ti_4_AlN_3_ MAX phase composite increases from 0.203 W m^−1^ K^−1^ at 950 °C to 0.303 W m^−1^ K^−1^ at 1250 °C as shown in Table 1. This suggests an increase in the material's ability to conduct heat as the sintering temperature increases, which could be due to changes in the microstructure such as grain boundary alterations. Similarly, the thermal diffusivity increases from 0.028 mm^2^ s^−1^ to 0.03 mm^2^ s^−1^ with the rise in sintering temperature. This indicates more heat spread through the material at higher sintering temperatures, showing increased thermal conductivity of the sample materials. The specific heat capacity increases dramatically from 0.2352 MJ m^−3^ K^−1^ to 9.0024 MJ m^−3^ K^−1^, suggesting that the material requires significantly more energy to raise its temperature at higher temperatures. This increase in specific heat could be related to the changes in the internal energy storage mechanisms of the material.

##### 3.2.1.2 Thermal conductivity of the Ti_3_AlC_2_ MAX phase composite

The thermal conductivity of the Ti_3_AlC_2_ MAX phase composite slightly increases from 0.208 W m^−1^ K^−1^ at 950 °C to 0.329 W m^−1^ K^−1^ at 1250 °C as shown in Table 1. This indicates a modest improvement in heat conduction at higher temperatures, which might be due to a more stable microstructure that enhances the material's heat transfer capabilities. The thermal diffusivity also shows a small increase from 0.03 mm^2^ s^−1^ to 0.04 mm^2^ s^−1^, suggesting a marginally faster heat spread through the material as temperature rises. The specific heat capacity increases from 0.2889 MJ m^−3^ K^−1^ to 8.6009 MJ m^−3^ K^−1^, indicating that the material needs more energy to increase its temperature at higher temperatures. However, this increase is less than that of the Ti_4_AlN_3_ MAX phase composite, reflecting different intrinsic properties and microstructural responses to temperature.

#### 3.2.2 Comparative analysis between the conductivity of Ti_4_AlN_3_ and Ti_3_AlC_2_ MAX phase composites

Upon analyzing the thermal properties of Ti_4_AlN_3_ and Ti_3_AlC_2_ MAX phase composites at 950 °C and 1250 °C, distinct trends are observed. At 950 °C, the Ti_4_AlN_3_ MAX phase composite demonstrates lower thermal conductivity (0.203 W m^−1^ K^−1^) than the Ti_3_AlC_2_ MAX phase composite (0.208 W m^−1^ K^−1^), signifying less heat conduction.^9^ However, at 1250 °C, the Ti_3_AlC_2_ MAX phase composite exhibits a higher thermal conductivity (0.329 W m^−1^ K^−1^) compared to the Ti_4_AlN_3_ MAX phase composite (0.303 W m^−1^ K^−1^), although both materials show increased heat conduction at elevated sintering temperature. Regarding thermal diffusivity, the Ti_4_AlN_3_ MAX phase composite shows a reduced value (0.028 mm^2^ s^−1^) at 950 °C compared to the Ti_3_AlC_2_ MAX phase composite (0.03 mm^2^ s^−1^). At 1250 °C, both materials have increased thermal diffusivity, with the Ti_3_AlC_2_ MAX phase composite (0.04 mm^2^ s^−1^) slightly outperforming the Ti_4_AlN_3_ MAX phase composite (0.03 mm^2^ s^−1^). Specific heat analysis reveals that the Ti_3_AlC_2_ MAX phase composite requires more energy to increase its temperature at both sintering temperatures, with specific heat values higher than those of the Ti_4_AlN_3_ MAX phase composite. Additionally, the specific heat increase with temperature is more pronounced in the Ti_4_AlN_3_ MAX phase composite, indicating significant changes in its thermal energy storage capacity.

### 3.3 Thermomechanical analysis (TMA)

**Fig. 5:**
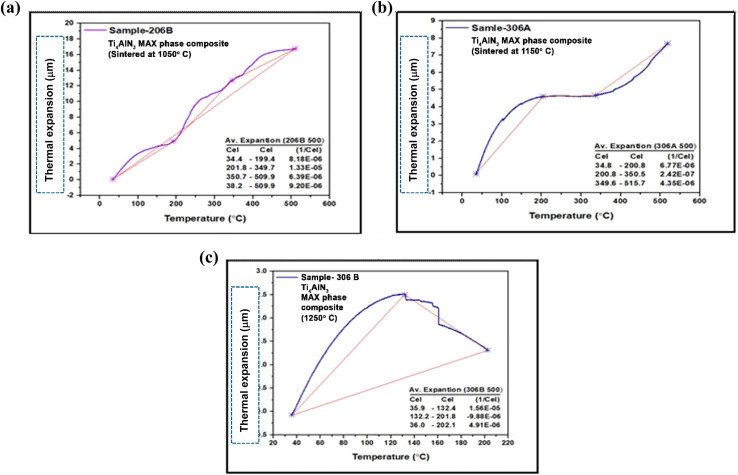
Coefficient of thermal expansion (μm m^−1^ °C^−1^) of the fabricated Ti_4_AlN_3_ MAX phase composite sample sintered at (a) 1050 °C, (b) 1150 °C and (c) 1250 °C.

Fig. 5(a)–(c) show the TMA graph of the Ti_4_AlN_3_ MAX phase composite sintered at three different temperatures 1050 °C, 1150 °C, and 1250 °C, respectively. The Ti_4_AlN_3_ MAX phase composite sintered at 1050 °C shows a coefficient of thermal expansion of 8.18 × 10^−6^ between 34.0 and 199.4 °C, 1.33 × 10^−5^ between 201.0 and 349.7 °C, and 6.39 × 10^−6^ between 350.7 and 509.9 °C, respectively. The overall coefficient of thermal expansion of the Ti_4_AlN_3_ MAX phase composite sintered at 1050 °C is 9.20 × 10^−6^ between 38.2 and 509.9 °C (Fig. 5(a)). Similarly, the Ti_4_AlN_3_ MAX phase composite sintered at 1150 °C shows a coefficient of thermal expansion of 6.77 × 10^−6^ between 34.8 and 200.8 °C, 2.41 × 10^−7^ between 200.8 and 350.5 °C, and 4.35 × 10^−6^ between 349.6 and 515.7 °C, respectively (Fig. 5(b)). Finally, the Ti_4_AlN_3_ MAX phase composite sintered at 1250 °C shows a coefficient of thermal expansion of 1.56 × 10^−5^ between 35.9 and 132.4 °C, −9.88 × 10^−6^ between 132.2 and 201.8 °C, and 4.91 × 10^−6^ between 36.0 and 202.1 °C, respectively (Fig. 5(c)). All these results are depicted in Table 2.

**Table 2:** Co-efficient of thermal expansion of the fabricated composite samples at different heating ranges

Sample name	Sintering temp. (°C)	Co-efficient of thermal expansion at different heating ranges (μm m^−1^ °C^−1^)			
		30–200 (°C)	201–350 (°C)	351–515 (°C)	30–515 (°C)
Ti_4_AlN_3_ MAX phase composite	1050 °C	8.18 × 10^−6^	1.33 × 10^−5^	6.39 × 10^−6^	9.20 × 10^−6^
	1150 °C	6.77 × 10^−6^	2.42 × 10^−7^	4.35 × 10^−6^	—
	1250 °C	4.91 × 10^−6^	−9.88 × 10^−6^	—	—
Ti_3_AlC_2_ MAX phase composite	1050 °C	7.47 × 10^−6^	1.48 × 10^−6^	3.33 × 10^−6^	—
	1150 °C	7.67 × 10^−6^	1.79 × 10^−7^	2.62 × 10^−6^	3.55 × 10^−6^
	1325 °C	6.99 × 10^−6^	1.63 × 10^−6^	−4.70 × 10^−7^	3.54 × 10^−6^

**Fig. 6:**
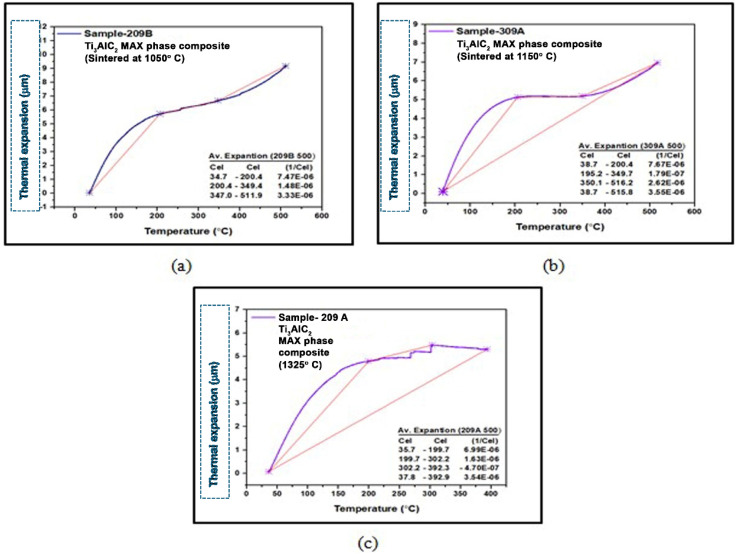
Coefficient of thermal expansion (μm m^−1^ °C^−1^) of the fabricated Ti_3_AlC_2_ MAX phase composite sample sintered at (a) 1050 °C, (b) 1150 °C and (c) 1325 °C.

Fig. 6(a)–(c) show the TMA graph of the Ti_3_AlC_2_ MAX phase composite sintered at three different temperatures 1050 °C, 1150 °C, and 1325 °C, respectively. Similarly, the Ti_3_AlC_2_ MAX phase composite sintered at 1050 °C shows a coefficient of thermal expansion of 7.47 × 10^−6^ between 34.7 and 200.4 °C, 1.48 × 10^−6^ between 200.4 and 349.4 °C, and 3.33 × 10^−6^ between 347.0 and 511.9 °C, respectively (Fig. 5(a)). The Ti_3_AlC_2_ MAX phase composite sintered at 1150 °C shows a coefficient of thermal expansion of 7.67 × 10^−6^ between 34.7 and 200.4 °C, 1.79 × 10^−7^ between 195.2 and 349.7 °C, and 2.62 × 10^−6^ between 350.1 and 516.2 °C, respectively. The overall coefficient of thermal expansion of the Ti_3_AlC_2_ MAX phase composite sintered at 1150 °C is 3.55 × 10^−6^ between 38.7 and 515.8 °C (Fig. 6(b)). Finally, the Ti_3_AlC_2_ MAX phase composite sintered at 1325 °C shows a coefficient of thermal expansion of 6.99 × 10^−6^ between 35.7 and 199.7 °C, 1.63 × 10^−6^ between 199.7 and 302.2 °C, and −4.70 × 10^−7^ between 302.2 and 392.3 °C, respectively. The overall coefficient of thermal expansion of the Ti_3_AlC_2_ MAX phase composite sintered at 1325 °C is 3.54 × 10^−6^ between 37.8 and 392.9 °C (Fig. 6(c)).

#### 3.3.1 Significance of experimental CTE of fabricated composites

The Ti_4_AlN_3_ MAX phase composite samples show different coefficients of thermal expansion (CTE) at various sintering temperatures. The CTE is critical in determining the material's dimensional stability under thermal cycling. The data suggest that sintering temperature significantly affects the thermal expansion properties. The CTE values of the sample sintered at 1050 °C vary across temperature ranges, with an overall CTE of 9.20 × 10^−6^ between 38.2 and 509.9 °C. A CTE of 6.77 × 10^−6^ in the heating range 34.8 to 200.8 °C of the sample sintered at 1150 °C indicates better thermal stability in this range. The sample sintered at 1250 °C shows a negative CTE in a specific range, indicating potential contraction and unusual behavior at these temperatures. Similarly, Ti_3_AlC_2_ MAX phase composite samples also exhibit varying CTEs based on sintering temperatures. Lower CTE values of the sample sintered at 1150 °C compared to the sample sintered at 1050 °C suggest better thermal stability. The sample sintered at 1325 °C shows a negative CTE in a specific range, indicating unusual behavior similar to that of the Ti_4_AlN_3_ MAX phase composite at higher sintering temperatures. Ti_4_AlN_3_ and Ti_3_AlC_2_ MAX phase composite samples show varying CTEs at different temperature ranges, indicating their thermal expansion properties are highly dependent on sintering temperatures. Both materials exhibit lower overall CTEs at higher sintering temperatures, suggesting increased thermal stability. The presence of negative CTEs in both materials at higher sintering temperatures indicates unusual thermal behavior, which could be due to phase transitions or changes in the microstructure. Higher sintering temperatures can lead to grain growth and changes in phase composition, affecting the CTE. For example, the negative CTE observed in Ti_4_AlN_3_ and Ti_3_AlC_2_ MAX phase composites (sintered at 1250 °C and 1325 °C respectively) in the temperature ranges 201 to 350 °C and 352 to 515 °C respectively might be due to phase transitions or specific microstructural changes that induce contraction. These transitions can lead to volumetric changes that result in contraction rather than expansion.^24,25^ The discovery of negative thermal expansion (NTE) materials, whereby the volume contracts instead of expanding upon heating, has potential engineering applications in thermal expansion control.^26,27^ Materials with a controllable coefficient of thermal expansion (CTE) can be achieved by forming composites between NTE materials and normal positive thermal expansion materials.^28^ Ti_4_AlN_3_ and Ti_3_AlC_2_ MAX phase composites belong to the MAX phases, characterized by their layered structure which can lead to anisotropic thermal expansion properties.^26^

### 3.4 Conclusion

Ti_4_AlN_3_ and Ti_3_AlC_2_ MAX phase reinforced B composites were successfully synthesized at varying sintering temperatures of 1050 °C, 1250 °C, and 1325 °C. The thermal analysis of Ti_4_AlN_3_ and Ti_3_AlC_2_ MAX phase composites indicates an enhancement in thermal conductivity, diffusivity, and stability with increasing sintering temperature. Remarkably, Ti_4_AlN_3_ exhibits superior thermal stability and a more controlled mass loss compared to Ti_3_AlC_2._ However, Ti_3_AlC_2_ demonstrates slightly enhanced thermal properties with increasing temperature, highlighting the suitability of both materials for high-temperature applications. At 1325 °C sintering, the MAX composites reach thermal stability limits up to 700 °C, with Ti_3_AlC_2_ showing higher weight gain (12.2%) than Ti_4_AlN_3_ (6.5%). The MAX composite structure undergoes gradual degradation while retaining a relatively stable thermal profile. Thermal conductivity analysis reveals that the composites depend on sintering temperature, with the Ti_3_AlC_2_ MAX phase composite exhibiting higher conductivity than the Ti_4_AlN_3_ MAX phase composite when sintered at an elevated temperature of 1250 °C.

Thermomechanical results indicate that sintering temperature has a significant impact on the thermal expansion properties. At higher sintering temperatures, both MAX phase composites demonstrate reduced overall CTEs, suggesting improved thermal stability. The CTEs at elevated sintering temperatures make these composites ideal for advanced electronics and aerospace use. Optimizing sintering conditions is crucial for enhancing material performance, ensuring they meet the demands of specific industrial applications effectively. Further exploration of reinforcement strategies and additive manufacturing techniques could also improve their structural integrity and versatility.

## Data availability

The data supporting this article is provided as part of the ESI.†

## Author contributions

Md. Shahinoor Alam: conceptualization, analysis, original draft, review and editing. Mohammad Asaduzzaman Chowdhury: supervision, analysis and funding acquisition. Md. Saiful Islam: review and editing. Md. Moynul Islam: investigation and data curation. Tasmina Khandaker: editing and data curation. M. A. Gafur: investigation and data analysis. Dipa Islam: investigation and software support.

## Conflicts of interest

The authors declare that this research paper does not have any financial and personal relationships with other people or organizations.

## Supplementary Material

NA-007-D5NA00063G-s001

NA-007-D5NA00063G-s002

NA-007-D5NA00063G-s003
